# Insulin Receptor Substrate-1 (IRS-1) Gly927Arg: Correlation with Gestational Diabetes Mellitus in Saudi Women

**DOI:** 10.1155/2014/146495

**Published:** 2014-02-17

**Authors:** Khalid Khalaf Alharbi, Imran Ali Khan, Zeinab Abotalib, Malak Mohammed Al-Hakeem

**Affiliations:** ^1^Department of Clinical Laboratory Sciences, College of Applied Medical Sciences, King Saud University, P.O. Box 10219, Riyadh 11433, Saudi Arabia; ^2^Department of Obstetrics and Gynecology, King Khalid University Hospital, College of Medicine, King Saud University, P.O. Box 60826, Riyadh 11555, Saudi Arabia

## Abstract

Pregnant women with gestational diabetes mellitus (GDM) and type 2 diabetes mellitus (T2DM) share a common pathophysiology associated with similar risk factors. Genetic variants used to determine the risk of developing T2DM might also be associated with the prevalence of GDM. The aim of the present study was to scrutinize the relationship between the G972R polymorphism of the insulin receptor substrate-1 (*IRS-1*) gene with GDM in the Saudi female population. This is a case-control study that monitored 500 Saudi women. Subjects with GDM (*n* = 200) were compared with non-GDM (*n* = 300) controls. We opted to evaluate rs1801278 polymorphism in the IRS1 gene, which plays a critical role in the insulin-signaling pathway. Genotyping was performed with the Polymerase Chain Reaction-Restriction Fragment Length Polymorphism (PCR-RFLP) method. The frequency of the rs1801278 polymorphism was significantly higher in women with GDM than in women with non-GDM (for TT + CT versus CC: *P* = 0.02). Additionally, there was a significant increase in the frequency of the Arg-encoding mutant allele from GDM to non-GDM (for T versus C: *P* = 0.01). Our results suggest that the rs1801278 polymorphism in the *IRS-1* gene is involved in the occurrence of GDM in the Saudi population.

## 1. Introduction

Gestational diabetes mellitus (GDM) is a growing health concern that usually appears during the latter half of pregnancy and is characterized by carbohydrate intolerance of variable severity [1].

The prevalence of GDM, which affects 2–22% of all pregnancies, varies across populations (e.g., ethnic groups) [2]. Risk factors for GDM include obesity, advanced maternal age, family history of type 2 diabetes mellitus (T2DM), past history of GDM, previous adverse pregnancy outcomes, and belonging to a high-risk ethnic group [3]. The genetic background of T2DM may also be a factor in GDM because ample evidence has demonstrated the presence of T2DM in women with GDM. Moreover, the prevalence of T2DM is relatively higher in mothers with GDM after pregnancy [4]. Epidemiological studies confirmed that the prevalence of GDM is in direct proportion to the prevalence of T2DM [5]. GDM and T2DM share a common genetic background, including glucose intolerance, insulin resistance, and impaired insulin secretion. However, association with similar risk factors and the genetic variants used to determine the risk of developing T2DM might also be associated with the prevalence of GDM [6, 7].

The insulin receptor substrate- (*IRS-*) 1 gene, located on chromosome 2q36, encodes a member of the *IRS* protein substrate family. IRS-1 is an endogenous substrate of the insulin receptor, plays a crucial role in the insulin signaling pathway, and is expressed in insulin-sensitive tissues. Several genetic polymorphisms of this gene and their effects on insulin action have already been identified [8]. A glycine-to-arginine substitution in codon 972 (Gly972Arg) of the *IRS-1 *gene (rs1801278) has been shown to be associated with a high prevalence of T2DM and GDM due to insulin resistance and impaired insulin secretion [6, 9]. The G972R polymorphism of *IRS-1*, which is located between two potential tyrosine phosphorylation sites involved in binding of the p85 subunit of PI-3 kinase, has previously been associated with T2DM [10]. However, a meta-analysis of 32 studies, including 12076 cases and 11285 controls, did not show the R972 variant to be significantly associated with T2DM [11].

We examined the *IRS-1* G972R polymorphism in 500 Saudi women: 200 GDM cases and 300 non-GDM subjects. The goal of this study is to determine whether the *IRS-1* G972R polymorphism affects GDM-afflicted Saudi women living in Saudi Arabia. In particular, our study focused on the G972R polymorphism, since this polymorphism is associated with T2DM, obesity, polycystic ovarian syndrome, and insulin resistance.

## 2. Materials and Methodology

### 2.1. Ethics

Informed consent was obtained from each patient involved in this study (E-12-675) and the Institutional Review Board of the College of Medicine, King Saud University (Riyadh, Saudi Arabia), granted ethical approval.

### 2.2. Sample Collection

Five milliliters of venous blood was collected; 3 mL of serum was used for biochemical analysis, and 2 mL was used for molecular analysis.

### 2.3. Study Population

In total, 500 pregnant women were included in this study. The subjects were from Riyadh, Saudi Arabia. Of these, 200 were unrelated patients with GDM who visited the primary health care outpatient clinic at King Khalid University Hospital (KKUH) in Riyadh. Women diagnosed with diabetes prior to pregnancy were excluded from this study. All pregnant women (*n* = 300) exhibiting normal glucose tolerance (NGT) were selected based on age and were designated as non-GDM/healthy controls.

### 2.4. Validation of GDM in Study Participants

All the pregnant women in this study were recruited after they visited the outpatient clinic at KKUH. Initially, the Glucose Challenge Test (GCT) was performed on all the pregnant women. Plasma glucose standards that exceeded 7.8 mmol/L were considered GCT-positive. Subsequently, the Oral Glucose Tolerance Test (OGTT) was performed. Before this test, pregnant women were advised to fast overnight in addition to three days of an unrestricted diet. Fasting plasma samples were drawn after 1, 2, and 3 h of administration of glucose to perform the 100 g OGTT test. In this study, Bhat et al. [12] abnormal values were followed (Table 1). If two or more values were abnormal, the patient was considered GDM-positive. Pregnant women with abnormal glucose tolerance tests were included in the study. NGT group members were considered non-GDM/healthy controls or normal controls.

### 2.5. Anthropometric and Biochemical Measurements

Anthropometric measurements were obtained by trained personnel at health care centers. Height and body weight were measured to the nearest 0.5 cm or 0.1 kg. Body Mass Index (BMI) was calculated as weight/height^2^ (kg/m^2^). Subjects with BMI > 30 kg/m^2^ were categorized as obese. Fasting and postprandial blood biochemical parameters included high density lipoprotein cholesterol (HDL-C), low density lipoprotein cholesterol (LDL-C), triglycerides (TG), and total cholesterol (TC). GCT and OGTT were measured by a commercially available clinical chemistry kit provided purchased from Konelab (Espoo, Finland).

### 2.6. Molecular Analysis

Genomic DNA was extracted from peripheral blood leukocytes using AccuVis DNA extraction kit (AccuVis Bio, UAE). DNA samples were stored at −80°C. Molecular analysis was performed at the facility of the Department of Clinical Laboratory Sciences, College of Applied Medical Sciences, King Saud University (Riyadh, Saudi Arabia).

### 2.7. Amino Acid Conversion at Gly972Arg

We used PCR-RFLP to amplify and genotype the nucleotide 972 polymorphism (i.e., rs1801278) in* IRS-1*, which is responsible for the glycine-to-arginine amino acid mutation. Amplification of the fragment was performed with forward primer 5′-CTTCTGTCAGGTGTCCATCC-3′ and reverse primer 5′-TGGCGAGGTGTCCACGTAGC-3′, which has been published by Pappa et al. [6]. Primers were synthesized by Bioserve Biotechnology, (Hyderabad, India) for PCR analysis. DNA was denatured at 95°C for 5 min and amplified by 35 cycles at 95°C for 30 s, 62°C for 30 s, 72°C for 45 s, and the final extension step at 72°C for 5 min. A total volume of 20 *μ*L reaction mixture contains 2 *μ*L of each primer (10 pmol), 7 *μ*L of sterile water and 10 *μ*L of 2× master mix (MgCl_2_, 10× Taq buffer, 10 unit of Taq DNA polymerase (Norgen Biotek corp, Canada)), and the 1 *μ*L template DNA used for amplification of the G972R polymorphism. PCR products were digested for 2 h with *Bst*NI (CC^↓^WGG) (New England Biolabs, Ipswich, MA) at 60°C (2.5 *μ*L of distilled water with 10 units of enzyme for 15 *μ*L PCR product and 2 *μ*L of 10× buffer in a final volume of 20 *μ*L) in nondenaturing Polyacrylamide Gel electrophoresis (PAGE) and visualized after silver staining. Three genotypes could be determined after electrophoresis: G972 homozygotes (159/81/23 bp), R972 (108/81/51/23 bp band), and G972R (159/108/81/51/23 bp) heterozygotes.

### 2.8. Analysis

Data are expressed as mean ± SD. Independent sample *t*-test was used to test GDM and non-GDM groups. Significance was set at *P* < 0.05. Allele and genotype frequency differences between GDM patients and non-GDM subjects were tested by chi-square analysis. Odds ratios (ORs) and 95% confidence intervals were calculated by multiple logistic regression analysis. Genotype and haplotype frequencies were used to identify departures from the Hardy-Weinberg equilibrium (HWE). Statistical analyses were performed with SPSS version 19.0 software. A *P* value of <0.05 was considered to be statistically significant.

## 3. Results

### 3.1. Clinical Characteristics

Contrasts between maternal demographic characteristics of GDM cases and non-GDM controls are listed in Table 2. The results illustrate that GDM subjects were similar to the controls, and anthropometric measurements including age, weight, height, and BMI remained the same (*P* > 0.05) in the GDM and non-GDM subjects. FBS, PPBG, GCT, OGTT, and lipid profile (TC, TG, HDL-C, and LDL-C) were the biochemical tests performed for all pregnant women and were found to be significant (*P* < 0.05). Family history of T2DM and GDM was significantly different between both groups (*P* < 0.05). Identified GDM women (90%) were prescribed a diet to maintain normal glucose values, whereas 10% of GDM women were using 4–8 units of insulin due to the diet failure.

### 3.2. Molecular Scrutiny for Gly972Arg

The distribution of Gly972Arg polymorphism in *IRS-1* is provided in Table 3. We observed that the frequencies of CC, CT, and TT genotypes of the rs1801278 SNP were statistically significant. IRS1 genotype frequencies of Gly972Gly, Gly972Arg, and Arg972Arg were 94.5%, 5%, and 0.5% in the GDM group and 98.3%, 1.7%, and 0% in the non-GDM, respectively. Allele frequencies of Gly and Arg were 97% and 3% for the GDM group and 99.2% and 0.008% for the non-GDM group, respectively. The TT genotype was absent in non-GDM groups. The minor allele frequencies of GDM and non-GDM were 3% and 0.008%, respectively (*P* < 0.05). Similarly, the frequency of CC, CT, and TT genotypes of the Gly972Arg SNP was comparable among the GDM and non-GDM (minor allele frequency; *P* = 0.01; OR = 3.7; 95% CI [1.3, 10.5]). There were significant differences in the frequencies of the genotype distributions of *IRS-1* Gly972Arg between the GDM group and non-GDM group (TT + CT versus CC: *P* = 0.02; OR-3.4; 95% CI [1.2, 10.1]). The odds ratio for any genotype of the Gly972Arg SNP was significantly correlated with the risk of developing GDM (*P* < 0.05) in the Saudi women monitored in this study. Genotype data is associated with HWE.

## 4. Discussion

King Saud University is one of the foremost institute in the Kingdom of Saudi Arabia as well as in an Arab countries involved deeply in the research with human and molecular medical genetics. This is a case-control study performed with 500 pregnant Saudi women, including 200 GDM and 300 non-GDM cases. We investigated the relationship between the *IRS-1* G972R gene and GDM in a Saudi population. Screening and identifying GDM are based on biochemical criteria with serum sample despite the progress of genetic screening methods. There is no genetic test to identify GDM in women during pregnancy. To the best of our knowledge, this is the first study conducted with pregnant Saudi women. In this study, we explored the effects of G972R of the *IRS-1* gene variants. The results of our study revealed that the variant allele Arg972 of the *IRS-1* gene is significantly associated with GDM.

Pregnancy is characterized by peripheral insulin resistance, which is compensated by an increase in insulin secretion to maintain glucose homeostasis [13]. GDM represents a heterogeneous disorder with both a genetic and an environmental component. The prevalence of GDM in Saudi Arabia is 18.7% [2]. According to additional studies, women with GDM were older and of higher parity and had a less favorable reproductive history than did nondiabetic pregnant women. The independent association between high parity and GDM was corroborated by previous reports [14]. Advanced maternal age is independently associated with an increased risk of GDM [2]. Several genetic polymorphisms of this gene have already been identified, as they affect insulin action [15]. The IRS-1 protein is a cytoplasmic molecule expressed in many insulin-sensitive tissues. It plays an important role in regulating the cellular effects of insulin [16]. Tyrosine phosphorylation of IRS-1 proteins initiates the downstream effects of insulin, including activation of phosphatidylinositol 3-kinase (PI3 K) and translocation of glucose transporter 4 [17]. The G972R polymorphism has also been associated with impaired *β*-cell function in NGT subjects as well as with reduced insulin content and impaired insulin secretion in isolated human islets [10]. Ample evidence suggests that susceptibility to GDM has a genetic component. Although no studies have evaluated the heritability of GDM, family studies indicate that GDM aggregates within families and is associated with a history of T2DM [18]. Genetic studies of T2DM and GDM further suggest that it is a multigenic disease in which common variants in multiple genes interact with environmental factors to cause the disease [19, 20]. Because of the striking parallels between GDM and T2DM, it is likely that GDM is also a multigenic disease related to T2DM. Thus, recent studies on the etiology of GDM have begun to evaluate the role of common polymorphisms in genes involved in T2DM predisposition. So far, genetic predisposition to GDM has been reported for variations in genes involved in insulin resistance and insulin secretion. However, no association could be replicated in different studies [21–23]. These discrepancies could be due to lack of power, given the small effect size of most common variants, or to ethnic heterogeneity among different populations.

Studies conducted by Pappa et al. [6] in the Greek population, with 148 women with GDM and 107 control subjects, and the results indicate that the G972R polymorphism of the *IRS-1* gene is strongly associated with increased susceptibility to GDM. Our results in the Saudi population are in complete agreement with the results reported by Fallucca et al. [13] and Pappa et al. [6], which indicate that the polymorphic allele R972 is significantly associated with *IRS-1* and may be involved in the development of GDM. In contrast, Shaat et al. [10] and Tok et al. [24] did not observe any association between the G972R polymorphism and GDM. Although no association was observed in these studies, Tok et al. [24] reported that the variant was associated with higher fasting glucose and insulin levels in women with GDM. Shaat et al. [10] concluded that IRS-1 protein levels are reduced in the adipose tissue of obese Scandinavian women with GDM. Our findings established that homozygosity for the G972R polymorphism, which was observed only in women with GDM, might indicate an increased risk for GDM in Saudi women.

In our study, 90% of the women were following a diet meant to regularize the levels of certain biochemical markers before pregnancy. Maintaining good nutrition during pregnancy, while consuming 2250–2500 calories daily, is essential. Physical exercise is also very important for GDM women, especially those identified as either overweight or obese. Insulin is the only medication/option for pregnant women who develop GDM during pregnancy. Insulin does not transfer to the baby through the placenta; however, the oral drug metformin (glyburide) can be used to circumvent this. Ten percent of the women in our study used insulin. The biochemical profiles of GDM subjects were significantly higher than the biochemical profiles of non-GDM subjects (*P* < 0.05).

In conclusion, our data indicate that the *IRS-1* G972R variant may facilitate the development of GDM. However, this variant was also associated with the baseline characteristics of the patients with GDM.

## Figures and Tables

**Table 1:** Diagnosis of GDM with a 100 g oral glucose tolerance test.

	mmol/L*	mg/dL**
Fasting	5.3 mmol/L	95 mg/dL
First hour	10.0 mmol/L	180 mg/dL
Second hour	8.6 mmol/L	155 mg/dL
Third hour	7.8 mmol/L	140 mg/dL

*mmol/L-millimolar/liter.

**mg/dL-milligram/deciliter.

**Table 2:** Demographic characteristics of the pregnant women.

	GDM Cases (*n* = 200)	Non-GDM (*n* = 300)	*P* value
Age (years)	32.43 ± 5.79	31.36 ± 6.02	0.55
Weight (Kg)	77.1 ± 13.34	74.85 ± 12.09	0.12
Height (m^2^)	158.51 ± 5.92	157.81 ± 5.31	0.08
BMI (kg/m^2^)	34.43 ± 4.68	33.36 ± 4.28	0.16
Mean gestational age	30.27 ± 5.77	NA	NA
FBS (mmol/L)	5.0 ± 0.93	4.5 ± 0.87	<0.001
PPBG (mmol/L)	6.8 ± 2.0	4.9 ± 1.8	0.001
GCT (mmol/L)	9.5 ± 1.8	6.3 ± 1.5	<0.001
OGTT (Fasting hour)	5.2 ± 1.18	4.5 ± 0.87	<0.001
OGTT (1st hour)	10.7 ± 1.8	8.0 ± 1.7	<0.001
OGTT (2nd hour)	9.2 ± 1.8	6.7 ± 1.6	<0.001
OGTT (3rd hour)	5.6 ± 1.7	4.5 ± 1.3	<0.001
TG (mmol/L)	2.3 ± 1.8	1.7 ± 0.98	<0.001
TC (mmol/L)	5.7 ± 1.2	5.2 ± 1.0	<0.001
HDL-C (mmol/L)	0.92 ± 0.38	0.64 ± 0.24	<0.001
LDL-C (mmol/L)	3.7 ± 0.93	3.7 ± 1.0	0.82
Family history of T2DM (*n* %)	120 (60%)	55 (18.3%)	<0.001
Family history of GDM (*n* %)	46 (23%)	13 (4.3%)	<0.001
*R*_*x*_ (Diet/Insulin)	180 (90%)/20 (10%)	NA	NA

All continuous variables represented by mean ± standard deviation. Independent sample *t*-test is done comparing GDM and non-GDM subjects. Categorical variables compared using chi-square analysis. A *P* < 0.05 was considered significant. NA: not applicable/not analyzed.

**Table 3:** Genotype and allele distribution of the *IRS1* (G972R) gene polymorphism for GDM and non-GDM.

*IRS*1 (rs1801278)	GDM *N* (%)	Non-GDM *N* (%)	Odds ratio^a^ (95% CI)	*P* value	Odds ratio^b^ (95% CI)	*P* value
*N*	200	300				
CC	189 (94.5)	295 (98.3)	Reference			
CT	10 (5.0)	5 (1.7)	3.1 (0.99, 9.2)	0.06*	2.9 (0.89, 9.9)	0.07
TT	1 (0.5)	0 (0.0)	—	—		
CT + TT	11 (5.5)	5 (1.7)	3.4 (1.2, 10.1)	0.02*	3.3 (1.0, 10.9)	0.04
C	388 (97)	595 (99.2)	Reference			
T	12 (3)	5 (0.08)	3.7 (1.3, 10.5)	0.01		

^a^Crude odds ratio (95% CI); ^b^odds ratio (95% CI) adjusted for age and BMI.

*Genotype and allele frequency distribution with GDM and Non-GDM subjects.
